# Genomic and immune determinants of resistance to daratumumab-based therapy in relapsed refractory multiple myeloma

**DOI:** 10.1038/s41408-024-01096-6

**Published:** 2024-07-19

**Authors:** Bachisio Ziccheddu, Claudia Giannotta, Mattia D’Agostino, Giuseppe Bertuglia, Elona Saraci, Stefania Oliva, Elisa Genuardi, Marios Papadimitriou, Benjamin Diamond, Paolo Corradini, David Coffey, Ola Landgren, Niccolò Bolli, Benedetto Bruno, Mario Boccadoro, Massimo Massaia, Francesco Maura, Alessandra Larocca

**Affiliations:** 1 Myeloma Division, University of Miami, Sylvester Comprehensive Cancer Center, Miami, FL USA; 2 Laboratory of Blood Tumor Immunology, Molecular Biotechnology Center “Guido Tarone”, Department of Molecular Biotechnology and Health Sciences, Università di Torino, Torino, Italy; 3 Division of Hematology, Azienda Ospedaliero-Universitaria Città della Salute e della Scienza di Torino, University of Torino, Torino, Italy; 4 Division of Hematology and Bone Marrow Transplant, Fondazione IRCCS Istituto Nazionale dei Tumori, Milan, Italy; 5 Hematology Unit, Fondazione IRCCS Ca’ Granda Ospedale Maggiore Policlinico, Milan, Italy; 6 Department of Oncology and Onco-Hematology, University of Milan, Milan, Italy; 7 European Myeloma Network, (EMN), Torino, Italy; 8 SC Ematologia, AO S. Croce e Carle, Cuneo, Italy

**Keywords:** Translational research, Cancer genomics, Myeloma, Immunotherapy

## Abstract

Targeted immunotherapy combinations, including the anti-CD38 monoclonal antibody (MoAb) daratumumab, have shown promising results in patients with relapsed/refractory multiple myeloma (RRMM), leading to a considerable increase in progression-free survival. However, a large fraction of patients inevitably relapse. To understand this, we investigated 32 relapsed MM patients treated with daratumumab, lenalidomide, and dexamethasone (Dara-Rd; NCT03848676). We conducted an integrated analysis using whole-genome sequencing (WGS) and flow cytometry in patients with RRMM. WGS before and after treatment pinpointed genomic drivers associated with early progression, including *RPL5* loss, APOBEC mutagenesis, and gain of function structural variants involving *MYC* and chromothripsis. Flow cytometry on 202 blood samples, collected every 3 months until progression for 31 patients, revealed distinct immune changes significantly impacting clinical outcomes. Progressing patients exhibited significant depletion of CD38-positive NK cells, persistence of T-cell exhaustion, and reduced depletion of regulatory T cells over time. These findings underscore the influence of immune composition and daratumumab-induced immune changes in promoting MM resistance. Integrating genomics and flow cytometry unveiled associations between adverse genomic features and immune patterns. Overall, this study sheds light on the intricate interplay between genomic complexity and the immune microenvironment driving resistance to Dara-Rd in patients with RRMM.

## Introduction

In the past two decades, there has been a notable transformation in the treatment of multiple myeloma (MM), with the development of novel and highly effective treatments. Notably, the incorporation of targeted immunotherapies, including the anti-CD38 monoclonal antibody (MoAb) daratumumab, has been one of the major breakthroughs in improving treatment response and clinical outcomes for both newly diagnosed (NDMM) and relapsed/refractory MM (RRMM) patients [1–9]. Despite these advancements, many patients still experience relapse, and the underlying mechanisms behind this clinical behavior remain largely unknown.

**Fig. 1:**
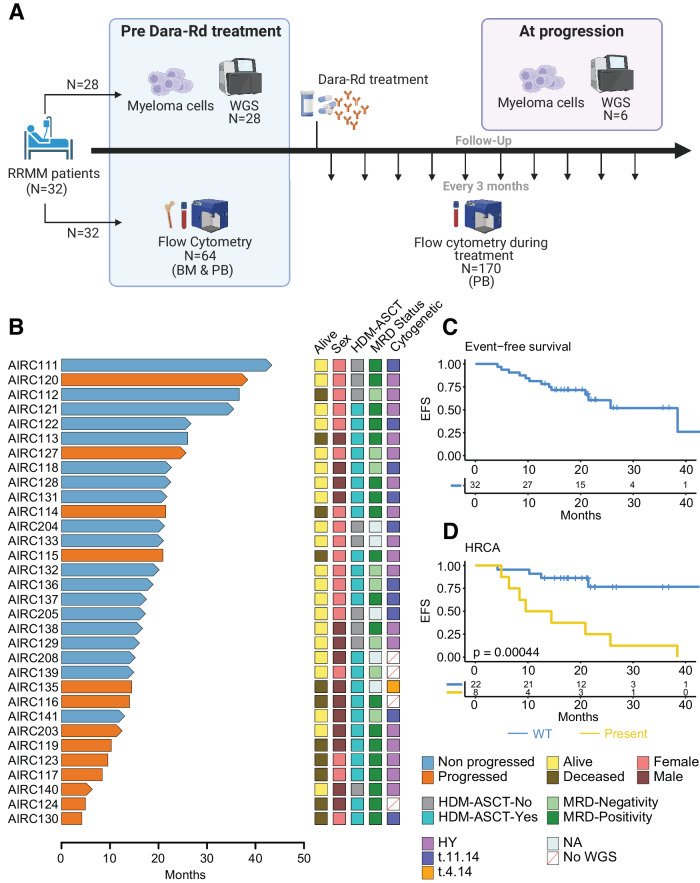
Study design and outcome in RRMM treated with anti-CD38 MoAb. **A** Study design. **B** Swimmer plot summarizing the clinical outcome of 32 patients with RRMM enrolled in this study. Arrows indicate patients that were still alive at the last follow-up; the no progressed patients (i.e., durable responders) are in light blue, while the progressors in orange. **C** Even free survival (EFS) Kaplan–Meier curve for the entire cohort. **D** EFS Kaplan–Meier curve comparing patients with and without the high-risk cytogenetic alterations [HRCA; t(4;14), t(14;16), del17p]; *P* values for (**C**, **D**) was generated with log-rank test. HY hyperdiploid.

Daratumumab, through binding to CD38 on malignant plasma cells, triggers direct cytotoxic effects and activates immune-mediated mechanisms. These include antibody-dependent cellular cytotoxicity (ADCC), complement-dependent cytotoxicity (CDC), antibody-dependent cellular phagocytosis (ADCP), apoptosis induced by cross-linking of CD38 on the target cells, and immunomodulatory effects via elimination of CD38-positive (pos) immunosuppressive cells in the immune tumor microenvironment (TME) [10–12]. Despite the efficacy of anti-CD38 antibody therapies, especially when combined with immunomodulatory agents (IMIDs) and proteasome inhibitors (PIs), there is a hypothesis that resistance of MM may result from a complex interplay between genomic factors within tumor cells and specific immune TME patterns. Ideally, this interplay could be investigated by integrating comprehensive genomic and immune data from MM patients treated with daratumumab. However, limited studies have explored resistance mechanisms to anti-CD38 MoAb in MM, with most focusing on either immune TME profiling or tumor biology using RNA/FISH-based data [2, 11, 13–15]. To comprehensively explore the interplay between the two compartments and track their changes over time, we performed a longitudinal collection of bone marrow (BM) and peripheral blood (PB) samples from a cohort of 32 patients with RRMM undergoing treatment with daratumumab, lenalidomide, and dexamethasone (Dara-Rd, ClinicalTrials.gov identifier NCT03848676). To elucidate genomic factors influencing treatment response or resistance, we conducted whole-genome sequencing (WGS) on CD138+ plasma cells at treatment initiation and disease progression. Concurrently, we employed multi-parametric flow cytometry to characterize the immune TME composition in both PB and BM samples from all 32 patients, prior to treatment initiation. Additionally, we investigated immune alterations over time by analyzing 202 PB samples collected every 3 months from the onset of treatment until disease progression for each patient (Fig. 1A). Our findings highlight that resistance to Dara-Rd in patients with RRMM stems from an interplay that involves distinct patterns of genomic complexity associated with specific immune profiles. Furthermore, it encompasses specific longitudinal changes in the immune cell composition of the TME. Notably, these patterns include the depletion of CD38 pos natural killer (NK) cells, the persistence of CD38 negative (neg) regulatory T cells (T-reg), and the endurance of exhausted T cells.

## Methods

Thirty-two relapsed/refractory MM patients were enrolled after informed consent and treated with Dara-Rd therapy (NCT03848676). A summary of key clinical and treatment data is available in Table 1 and Supplemental Table 1. The WGS was performed at the San Raffaele Institute. Samples were run on NovaSeq 6000 in a 2 × 150 bp paired end (Illumina). The pre-processing steps and the variant file generation have been performed at San Raffaele Institute using the nf-core/sarek pipeline (https://github.com/nf-core/sarek). Flow cytometry was performed at the Center for Experimental Research and Medical Studies (CeRMS), A.O.U. Città della Salute e della Scienza (Turin) to investigate the immune composition at baseline and post Dara-Rd treatment in BM and PB samples of 32 patients [16, 17]. Details about the WGS and flow cytometry can be found in Supplemental Methods. The comparison tests for linear features between different categorical groups were performed using a two-sided Wilcoxon test. Association of categorical variables with event-free survival (EFS) were implemented in an univariable fashion, using Kaplan–Meier curves and a log-rank test. EFS was measured from the date of start of treatment to the date of progression. Deaths from causes other than progression were censored.

**Table 1:** Patients’ characteristics.

Characteristic	*n* (%)	Progressors, *n* (%)	Durable responders, *n* (%)	*P* value	FDR
Total	32	13 (40.6%)	19 (59.4)	–	–
Age					
<65 years	12 (37.5%)	7 (21.9%)	5 (15.6%)	0.15	0.35
>=65 years	20 (62.5%)	6 (18.8%)	14 (43.8%)		
Gender					
Female	18 (56.2%)	7 (21.9%)	11 (34.4%)	1	1
Male	14 (43.8%)	6 (18.8%)	8 (25%)		
Previous therapy exposure					
HDM + ASCT	23 (71.9%)	11 (34.4%)	12 (37.5%)	0.25	0.404
Bortezomib	27 (84.4%)	12 (37.5%)	15 (46.9%)	1	1
Ixazomib	2 (6.3%)	0	2 (6.3%)	0.5	0.69
Thalidomide	11 (34.4%)	4 (12.5%)	7 (21.9%)	0.71	0.89
Lenalidomide	3 (9.4%)	1 (3.1%)	2 (6.2%)	1	1
Refractory status					
Bortezomib	7 (21.9%)	5 (15.6%)	2 (6.2%)	0.1	0.26
Thalidomide	0	–	–	–	–
Lenalidomide	1 (3.1%)	0	1 (3.1%)	1	1
ISS^a^					
I	12 (37.5%)	1 (3.1%)	11 (34.4%)	0.008	0.084
II	9 (28.1%)	5 (15.6%)	4 (12.5%)	0.25	0.404
III	6 (18.8%)	5 (15.6%)	1 (3.1%)	0.02	0.084
NA	5 (15.6%)	2 (6.2%)	3 (9.3%)	–	–
Cytogenetics					
Hyperdiploid	17 (53.1%)	9 (28.1%)	8 (25%)	0.17	0.36
t(11;14)	10 (31.2%)	1 (3.1%)	9	0.02	0.084
t(4;14), t(14;16), del17p	8 (25%)	8 (25%)	0	0.0001	0.002
Gain or amp1q	16 (50%)	8 (25%)	8 (25%)	0.47	0.66
Best response after Dara-Rd					
CR & sCR	9 (28.1%)	1 (3.1%)	8 (25%)	0.05	0.15
VGPR	12 (37.5%)	4 (12.5%)	8 (25%)	0.72	0.89
PR	8 (25%)	6 (18.8%)	2 (6.2%)	0.04	0.14
SD	1 (3.1%)	1 (3.1%)	0	0.41	0.62
NA	2 (6.3%)	1 (3.1%)	1 (3.1%)	–	–
MRD status by NGF					
Negative	7 (21.9%)	1 (3.1%)	6 (18.8%)	0.19	0.36
Positive	19 (59.4%)	11 (34.4%)	8 (25%)	0.03	0.084
NA	6 (18.8%)	1 (3.1%)	5 (15.6%)	–	–

*HDM-ASCT* high-dose melphalan and autologous stem cell transplant, *MRD* minimal residual disease, *NGF* next-generation flow.

^a^At diagnosis.

## Results

### Patient cohort

To delineate the genomic and immune factors contributing to resistance against Dara-Rd, we analyzed data from WGS and flow cytometry in a longitudinal cohort of 32 patients with RRMM enrolled in the NCT03848676 observational study (Fig. 1A). Clonal evolution analysis also included two patients: one treated with daratumumab single agent, and the other with daratumumab, bortezomib, and dexamethasone (Dara-Vd). The median age was 68 (range 46–80); 18 (56.2%) were women. The median number of prior treatments was 1 (range 1–3), with 23 (71.8%) patients previously exposed to high-dose melphalan chemotherapy followed by autologous stem cell transplantation (HDM-ASCT; Fig. 1B). Before starting Dara-Rd, 97% (*N* = 31) and 50% (*N* = 16) of patients were previously exposed to PI and IMIDs, respectively. After a median follow-up of 18.2 months (range 4.2–43.4), 40.6% (*N* = 13) patients experienced progression (i.e., progressors). Patients that did not experience progression were defined as “durable responders”. Overall, 8 (25%) patients achieved minimal residual disease (MRD) negativity by next-generation flow cytometry (Fig. 1B) [17]. The median event-free survival (EFS) was 38.4 months (Fig. 1C). Two patients (AIRC112 and AIRC113) died due pulmonary sarcoma and COVID pneumonia without relapse. Considering all the relapsed patients 9 out of 13 (69.2%) died due disease progression. Combining WGS and FISH data, the presence of at least one high-risk cytogenetic alteration (HRCA) [i.e., del17p and/or t(4;14) and/or t(14;16)] was observed in 8 out of 30 patients (26.7%; FISH and WGS data was missing in 2 patients) and it was associated with poor EFS (*P* = 0.00044; log-rank test; Fig. 1D and Supplemental Tables 1 and 2) [14]. We did not see an influence of other key clinical and serological features on the clinical outcome (Supplemental Table 2). Demographics, disease characteristics, and response to Dara-Rd for all patients are summarized in Table 1 and Supplemental Table 1.

### Genomic determinant of resistance and progression to Dara-Rd

To investigate the intrinsic genomic drivers associated with resistance to Dara-Rd, we performed WGS to profile the genomic landscape in BM samples at baseline and at progression. Four samples out of 32 failed the sequencing due to low cellularity or cancer cell fraction and therefore were excluded. The median coverage for the 28 cases that successfully completed WGS was 70× (Supplemental Table 3). The median single base substitution (SBS) burden was 5800 (range 1808–17,411). There was no difference in total mutational burden between progressors and durable responders. To assess the clinical implications of major somatic drivers in MM, we utilized an extensive catalog of established myeloma genomic drivers derived from large cohorts of NDMM patients, that encompasses mutations, mutational signatures, recurrent aneuploidies, and canonical translocations (Supplemental Methods and Supplemental Table 4) [18, 19]. Among the catalogs of 90 nonsynonymous MM driver mutations [18–20], we found 31 mutated genes in the patients with RRMM treated with Dara-Rd (Supplemental Fig. 1A). Genes in MAPK pathway (*NRAS*, *KRAS* and *BRAF*) were the most frequently mutated, with 61% of RRMM cases (treated with Dara-Rd) carrying at least one mutation in one of these genes (Supplemental Fig. 1A). Nevertheless, neither MAPK pathway nor any other mutations in MM driver genes predicted poor anti-CD38 MoAb outcomes (Supplemental Table 5). Finally, we did not find any mutation involving *CD38* gene either before or after treatment, but only a large deletion in one patient [21].

**Fig. 2:**
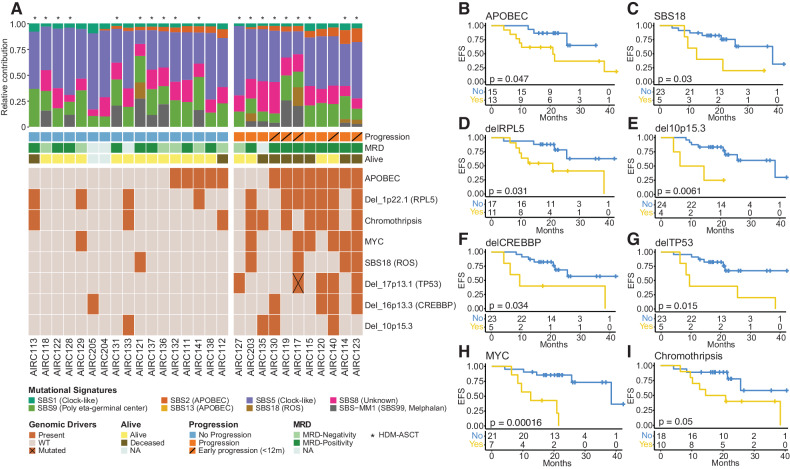
Impact of genomic alterations on clinical outcome in RRMM treated with Dara-Rd. **A** A heatmap showing all the genomic alterations associated with progression after Dara-Rd among patients with available WGS data (*N* = 28); at the top, the bar plot with the relative contribution per each SBS mutational signature. Minimal residual disease (MRD) was tested using Euroflow [17]. WT wild type, HDM-ASCT high-dose melphalan and autologous stem cell transplant, ROS radical oxygen stress. **B**–**I** Kaplan–Meier curves showing the impact of APOBEC (**B**), SBS18 (**C**), deletion 1p22.1 (*RPL5*)*;* **D**), deletion 10p15.3 (**E**), deletion 16p13.3 (*CREBBP*); **F**), deletion 17p13.1 (*TP53*); **G**), structural variants on *MYC* (*MYC*, *PTV1*, and *NSMCE2*); **H**) and presence of chromothripsis (**I**) on event-free survival (EFS); *P* value is calculated with log-rank test.

To investigate underlying mutational processes involved in these cases, we performed a mutational signature analysis (Supplemental Methods) [22, 23]. We identified seven SBS signatures involved in our cohort of RRMM: the clock-like aging signatures (SBS1 and SBS5), APOBEC mutational activity (SBS2 and SBS13), germline center associated polymerase eta (SBS9), radical oxygen damage (SBS18), SBS8, and SBS-MM1 (i.e., SBS99; melphalan mutagenesis; Fig. 2A and Supplemental Table 6) [19, 23–30]. SBS-MM1/SBS99 mutational signature was identified in 14 out of 19 (74%) patients who received HDM-ASCT. The lack of SBS-MM1/SBS99 in 5 patients can be explained through the single-cell expansion model as previously described [31]. Specifically, it has been shown that distinct chemotherapy agents promote their mutational activity by introducing a unique catalog of mutations in each exposed single cell [25, 31, 32]. These mutations can be detected by bulk WGS only if a single tumor cell exposed to chemotherapy expands, taking clonal dominance. In contrast, if the cancer progression is driven by multiple clones originating from different single cells exposed to chemotherapy, the chemotherapy-induced mutational signature will not be detectable because each clone harbors different catalogs of unique chemotherapy-related variants. Interestingly, two patients (AIRC119 and AIRC128) exhibited two distinct set of melphalan-related variants detected in both subclonal and clonal variants. This scenario was explained by the patients’ exposure to tandem HDM-ASCT. Among the mutational signatures identified in our cohort, high APOBEC contribution was associated to higher rate of progression (*P* = 0.03 estimated using Fisher exact test) and short EFS (*P* = 0.047; log-rank test; Fig. 2B and Supplemental Table 6). APOBEC has been shown to be one of the strongest prognostic markers for poor outcomes in NDMM treated with and without daratumumab-based regimens [18, 24, 26, 33]. These findings suggest that Dara-Rd treatment cannot fully counteract the negative prognostic impact of high APOBEC mutational activity. Among the other SBS signatures, the presence of SBS18, known to be caused by reactive oxygen species damage and associated with resistance to CART in diffuse large B-cell lymphoma [34], was associated with shorter EFS, with 4 out of 5 patients (80%) progressing after anti-CD38 MoAb combination treatment (*P* = 0.03 estimated using log-rank test; Fig. 2C and Supplemental Table 6). Finally, high SBS9 showed favorable outcomes, most likely due to the known inverse correlation between APOBEC and SBS9 (*P* = 0.041; log-rank test; Supplemental Table 6) [25, 33].

Next, we investigated the clinical impact of recurrent MM aneuploidies and structural variants (Supplemental Fig. 1B and Supplemental Methods) [18, 35]. Different deletions were associated with shorter EFS. Specifically, deletion of 1p22.1 (*RPL5*) was associated with shorter EFS (*P* = 0.031; log-rank test; Fig. 2D). Mono and bi-allelic loss of *RPL5* have been associated with poor response in both Dara and non-Dara-based regimens [33, 36]. In addition to *RPL5*, patients with loss of 10p15.3, 16p13.3 (*CREBBP*) and *TP53* were characterized by shorter EFS (*P* = 0.0061, *P* = 0.034 and *P* = 0.015; log-rank test, respectively; Fig. 2E–G). Since the identified aneuploidies 16p13.3 and 10p15.3 are genomic event not extensively characterized in the literature, we investigated their biological impact using paired WGS and RNA sequencing data from 705 patient enrolled in the CoMMpass study (https://themmrf.org). Running a gene set enrichment analysis (GSEA), we found that patients harboring one of these two alterations (versus wild-type) were enriched for *E2F, MYC* targets and *G2M* checkpoint pathways (Supplemental Fig. 2). These pathways were also found to be enriched in patients with RRMM that progressed after Daratumumab, Carfilzomib, Lenalidomide and dexamethasone at first relapse (Kydar study) [13, 33]. These data suggest that 16p13.3 and 10p15.3 deletions can contribute to the deregulation of distinct high-risk pathways associated with resistance to Dara-Rd treatment.

Thanks to WGS resolution, we were able to investigate the clinical impact of structural variants (SVs) and complex events in patients treated with Dara-Rd (Supplemental Fig. 1B) [35]. SVs causing *MYC* gain of function were associated with shorter EFS, similar to what was observed in NDMM treated with Dara-KRd (i.e., *NSMCE2* deletions, *MYC* and *PVT1* events; *P* = 0.00016; log-rank test; Fig. 2H) [33]. Among complex events [18, 35], the presence of chromothripsis, a well-recognized adverse prognostic factor marker in NDMM, and it was found in 36% (10/28) of patients, with 7 experiencing progression (*P* = 0.05; log-rank test; Fig. 2I) [35]. Genomic events associated with shorted EFS were not differentially distributed between early (EFS < 12 months) and late progressors. Despite the limitations in our sample size, we conducted a multivariate analysis using Cox proportional hazard model with the genomic drivers associated with poor outcomes, revealing that the mutational process associated with reactive oxygen species damage (SBS18) and SVs involving *MYC* were significantly independent of HRCA and ISS classification (*P* = 0.01217 and *P* = 0.00581, respectively; Supplemental Table 7 and Supplemental Methods). Overall, these data suggest that distinct genomic events and patterns of genomic complexity are associated with resistance to Dara-Rd treatment.

### Clonal evolution post Dara-Rd treatment

**Fig. 3:**
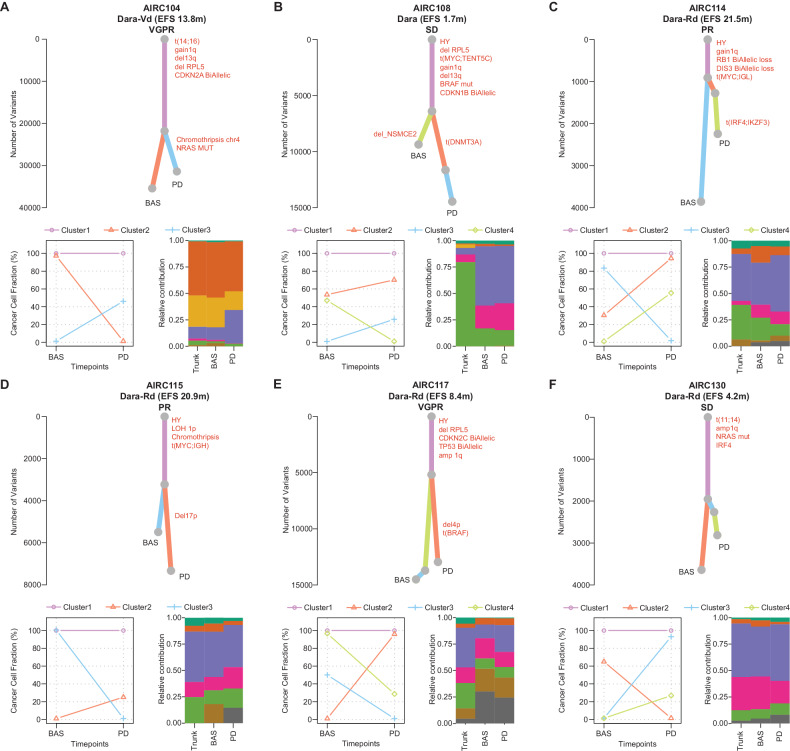
Clonal evolution of patients with RRMM treated with daratumumab-based treatment. Top of each panel: the phylogenetic tree with the trunk in violet and the branches in salmon, light blue, and light green. The *y* axis is the number of single nucleotide variants. At the bottom left side, the cancer cell fraction (CCF) scatter-plot per each cluster at baseline (BAS) and progression (PD). In the bottom right side, the bar plot with the mutational signature relative contribution for each cluster. The mutational signature color legend is the same of the Fig. 2. HY hyperdiploid.

To reconstruct the genomic evolution from baseline to progression after Dara-based treatment, we analyzed WGS data from four patients with longitudinal paired samples collected before treatment and at progression. In addition, we included two patients treated with daratumumab as a single agent or in combination with bortezomib and dexamethasone (Dara-Vd), with available WGS data before and after treatment. Within these six patients, we identified patterns of branching evolution in all cases, with 50% experiencing a complete clonal shift (Fig. 3A–F). The presence of clonal shift was independent of the patients’ EFS. Although we did not observe any recurrent genomic events, we did detect distinct and intriguing events that were undetectable at baseline but became dominant upon progression. In the patient AIRC104 the progression was driven by a clone with a previously undetectable chromothripsis event involving chromosome 4, but not *CD38* (Fig. 3A). In AIRC114 and AIRC115 the progression was driven by clone carrying translocations between chromosome 4 and 17 involving *IRF4* and *IKZF3* genes, and a large deletion of *TP53*, respectively (Fig. 3C, D). In AIRC117 at progression, the dominant clone had a deletion of chromosome 4p involving *CD38* undetectable at baseline (Fig. 3E) [21].

### Association between immune profiles at baseline and clinical outcomes

To explore the immune composition and its impact on clinical outcomes, BM and PB samples collected at baseline from 32 patients were investigated by flow cytometry (Supplemental Methods; Supplemental Table 8). In line with what was previously shown, both the relative and absolute number of immune cells were highly correlated between BM and PB, with 77% of cell types having Spearman coefficient equal or greater than 0.50 (*P* < 0.001; Supplemental Fig. 3 and Supplemental Table 9) [37, 38]. Next, we investigated the association between distinct immune patterns and MM response to Dara-Rd. First, we characterized the immune cell landscape with particular attention to CD38 pos immune cells and pattern of immune exhaustion. This analysis revealed that most myeloid-derived suppressor cells (MDSC, CD33 + CD11b + ), monocytes (CD14 + ), and natural killer (NK, CD56 + ) cells were CD38 pos in both BM and PB samples (Supplemental Fig. 4A). Noticeably lower counts and proportions of CD38 pos cells were observed in conventional CD4 + T-helper cells and CD8 + cytotoxic T cells, Vγ9 Vδ2 T cells, B-reg (CD38^hi^ CD19 + CD24 + ) [10], and T-reg (identified by CD4 + CD25 + + CD127lo and CD4 + CD25 + + FOXP3 + phenotypes) (Supplemental Fig. 4A). Despite monocytic-MDSCs (Mo-MDSCs, CD33 + CD14 + CD11b + ) exhibited a higher CD38 pos expression compared to granulocytic-MDSCs (Gr-MDSCs, CD33 + CD14 - CD11b + ) at the baseline in both BM and PB (Supplemental Fig. 4B), they were less prevalent than Gr-MDSCs (Supplemental Fig. 4C). Furthermore, at baseline all patients showed high prevalence of CD38 pos classical (CD14 + + CD16 -) and intermediate (CD14 + + CD16 + ) monocytes, and the classical subgroup was the most predominant compared with intermediate and nonclassical monocytes (Supplemental Fig. 4D, E). In addition, at baseline in BM samples the fraction of CD38 pos was higher among cytotoxic NK cells (CD56 + CD16 + ) than proliferative NK cells (CD56 + CD16 -) (Supplemental Fig. 4F). Across the whole BM immune populations, the proportion of cytotoxic NK cells was less abundant than the proliferative NK cells (Supplemental Fig. 4G).

**Fig. 4:**
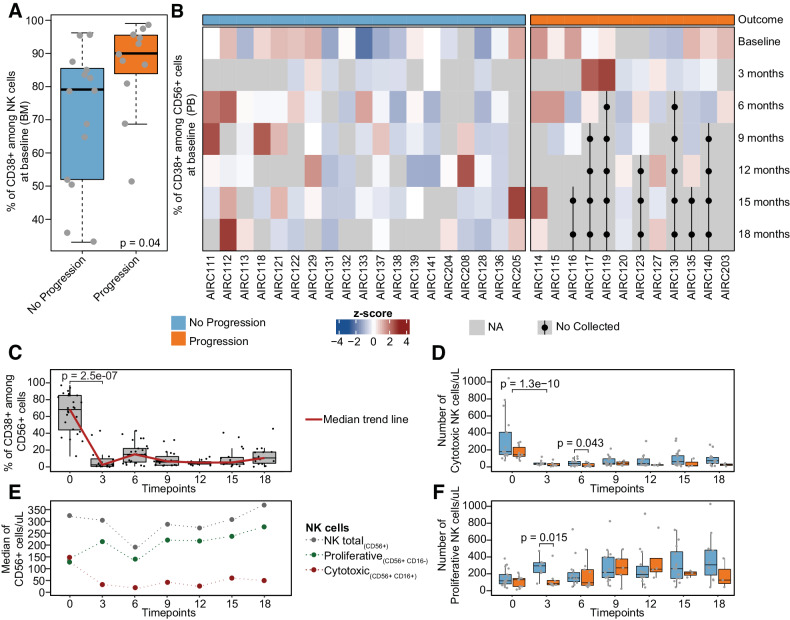
Daratumumab immunomodulation on natural killer cells. **A** Boxplot showing the percentage of CD38 pos among NK cells (CD56 pos). Light blue indicates durable responders, orange the progressors. *P* value was calculated with Wilcoxon test two-sided. **B** Heatmap showing CD38 pos in CD56 pos NK cells z-scores per each patient at seven different time points. Dots and black lines indicate that the sample was not collected at that time point, as the patient has previously progressed; one patient (AIRC124) was excluded because several samples were not collected at different time points. **C** Boxplots displaying the percentage of CD38 pos among CD56 pos NK cells over time. The red line connects the median value at each time point. **D** Boxplots showing the absolute number of cytotoxic NK cells over time. **E** Smooth plot presenting different NK cells patterns over time. **F** Boxplots showing the number of proliferative NK cells over time. **C**–**F** The *P* values were estimated using Wilcoxon test two-sided. The full list of all *P* values is reported in Supplemental Tables 11 and 12.

Next, we went to investigate the impact of the BM and PB immune composition on clinical outcomes. At baseline, patients who experienced progression after Dara-Rd treatment showed an enrichment of CD38 pos NK cells (*P* = 0.04 in the BM and *P* = 0.05 in the PB estimated by Wilcoxon test; Fig. 4A and Supplemental Fig. 5A, B). This was also confirmed by measuring the mean fluorescence intensity (MFI); the CD38 expression on NK cells was higher at baseline in progressors compared with durable responders (*P* = 0.024 in the BM estimated by Wilcoxon test; Supplemental Fig. 5C). Furthermore, at baseline, progressor after Dara-Rd exhibited an enrichment of CD38 pos Mo-MDSC and Gr-MDSC cells compared to durable responders (*P* = 0.033 and *P* = 0.01 estimated by Wilcoxon test, respectively in the BM only; Supplemental Fig. 5A). Interestingly, at baseline, patients who progressed were enriched in higher levels of exhausted T cells, such as TIM3 + cytotoxic T cells and TIM3 + helper T cells (*P* = 0.0089 and *P* = 0.0031 respectively; Supplemental Fig. 5D–G) in PB, and lower presence of helper T cells (*P* = 0.015; Supplemental Fig. 5H, I) in BM. Moreover, at baseline, a significant enrichment of classical and intermediate monocytes was identified in progressed patient PB samples (*P* = 0.039 both; Supplemental Fig. 5J, K). These data suggest Dara-Rd efficacy in patients with RRMM, is influenced by distinct patterns of immune composition at baseline, particularly high CD38 pos immune cells, exhausted T cells, and reduced helper T cells in the BM and/or PB.

### Immune modulation induced by Dara-Rd treatment over time

Given the concordance observed between BM and PB samples, we longitudinally assessed how Dara-Rd treatment influenced the immune system’s composition over time. This involved analyzing 202 PB samples collected at various time points from 31 study-enrolled patients. (Supplemental Table 8). One sample was excluded (AIRC124) due to lack of longitudinal samples after Dara-Rd treatment. As expected, both CD38 pos tumor and normal cells were rapidly depleted after exposure to daratumumab (Supplemental Fig. 6A and Supplemental Table 10) [15, 33, 39]. Among the main immune cell populations, a significant depletion was observed among cytotoxic and CD38 pos NK cells immediately after Dara-Rd treatment (*P* < 0.0001 estimated by Wilcoxon test at 3 month; Fig. 4B–D). In contrast, we observed an expansion of proliferative NK cells (Fig. 4E and Supplemental Table 11). This expansion was driven by CD38 neg proliferative NK cells that were not depleted by the daratumumab. Interestingly, the expansion of proliferative NK cells was significantly higher in durable responders compared to progressors (*P* = 0.015 estimated by Wilcoxon test; Fig. 4F and Supplemental Table 12). Durable responders were also characterized by a significant expansion of cytotoxic NK cells at 6 months (*P* = 0.043 estimated by Wilcoxon test, Fig. 4D and Supplemental Table 12). Consistent with earlier research, this data underscores the critical role of NK cells in the efficacy of anti-CD38 MoAb-based treatments [15, 33, 39]. Furthermore, these findings suggest a scenario in which daratumumab facilitates the fratricide depletion of CD38 pos NK cells, potentially constraining the subsequent availability of immune effector cells in the fight against myeloma cells. Because our flow cytometry panel utilized a CD38 multiepitope monoclonal antibody, the reduction in CD38-positive NK cells over time is likely due to depletion rather than CD38 epitope masking (see Supplemental Methods). This antibody effectively detected CD38, even when bound to daratumumab, using a distinct binding site, thereby avoiding false negative identification of CD38-masked NK cells. To further validate our observations, we analyzed CITEseq data from 20 NDMM patients undergoing Dara-KRd treatment [33]. We assessed NK cell count, CD38 expression, and protein levels in NK cells pre and post dara-based therapy. Our analysis revealed a significant reduction in NK cell count following Dara-KRd treatment (*P* = 0.0068; Supplemental Fig. 6B). Moreover, CD38 mRNA levels in NK cells exhibited a decrease post-treatment (*P* = 0.002; Supplemental Fig. 6C), which corresponded to a decrease in protein concentration (*P* = 0.0266; Supplemental Fig. 6D). Overall, these findings suggest that NK cells after Dara-based therapy exhibit decreased CD38 expression, rather than CD38 presence with daratumumab attached.

Next, we investigated the immune dynamics of monocyte cells and the classical (CD14 + + CD16 -), intermediate (CD14 + + CD16 + ), and nonclassical (CD14 + CD16 + +) subgroups. The longitudinal analysis showed a significant reduction of CD38 pos monocytes at 3 months after Dara-Rd treatment (*P* = 0.0001 estimated by Wilcoxon test), and the reduction was particularly relevant among the classical and intermediate subgroups (*P* = 0.0005 and *P* = 0.03 estimated by Wilcoxon test, respectively; Supplemental Fig. 7A and Supplemental Table 11). Interestingly, after being depleted at 3 months, the CD38 pos intermediate monocytes experienced a significant expansion after 12 months of therapy (*P* = 0.0042 estimated by Wilcoxon test; Supplemental Fig. 7A and Supplemental Table 11). In addition, independently from the CD38 status, reduction of intermediate and nonclassical monocyte subgroups was observed at 3 months after Dara-Rd treatment (*P* = 0.0036 and *P* = 0.0001 estimated by Wilcoxon test; Supplemental Fig. 7B, C and Supplemental Table 11).

**Fig. 5:**
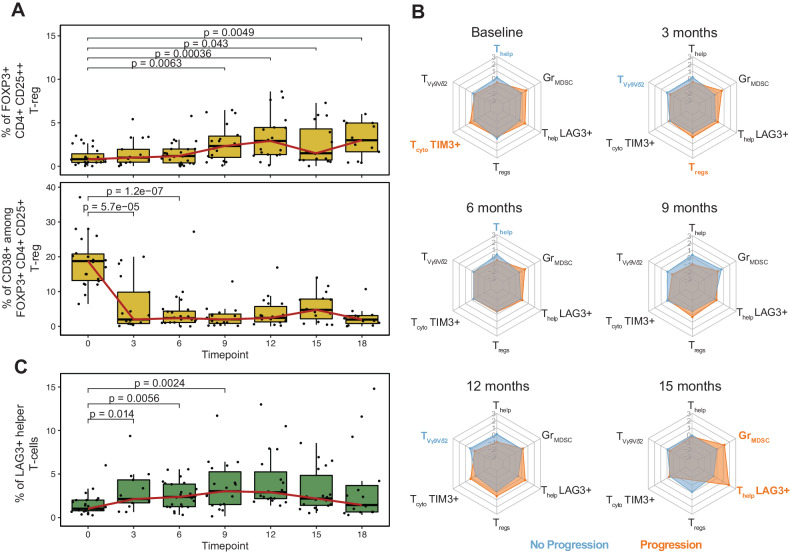
Regulatory and exhausted T cells change over time. **A** Boxplot showing the percentage of FOXP3 T-regs among all T-CD4 (top) and the percentage of CD38 pos among FOXP3 T-regs (bottom). The red line connects the median value at each time point. *P* values were estimated using Wilcoxon test two-sided. **B** Radar plots for each time point reporting the contribution of each cell types. The gray network indicates the z-scores values (from −3 to +3), each corner represents an immune cell type; the values represented are the median values of the z-scores for each cell type; the light blue hexagons refer to the values of durable responders, while the orange ones refer to the progressors. Bold and colored cell type names indicate that the specific cell type was significantly enriched at that time point. z-scores were computed using the relative values. T_reg_ = FOXP3 + T-reg. All *P* values were estimated using Wilcoxon test two-sided. **C** Boxplot displaying the percentage of LAG3 helper T cells among all T-CD4 over time. The red line connects the median value at each time point. *P* values were estimated using Wilcoxon test two-sided. The full list of all *P* values is in the Supplemental Table 11.

Finally, we investigated the composition and clinical implications of T-reg and of patterns of T-cell exhaustion. While the proportion of T-reg (i.e., CD4 + CD25 + + FOXP3 + ) over both the total number of immune cells and the T-CD4 experienced a significant expansion over time, in particular after 9 months across all patients (Fig. 5A and Supplemental Table 11), the CD38 pos T-reg experienced a significant and sustained depletion immediately after the first cycles of Dara-Rd (*P* < 0.0001 estimated by Wilcoxon test, Fig. 5A, Supplemental Fig. 7D, and Supplemental Table 11). While the proportion of CD38 pos T-reg was not significantly different between durable responders and progressors, the latter group had a higher proportion of all T-reg (i.e., CD38 pos and CD38 neg) over the total number of T-CD4 at 3 months compared to durable responders (*P* = 0.05 estimated by Wilcoxon test; Fig. 5B; Supplemental Table 12). In terms of T-cell exhaustion, we found an increase in LAG3 + T cells following Dara-Rd treatment, particularly LAG3+ helper T cells (*P* = 0.014 estimated by Wilcoxon test; Fig. 5C; Supplemental Table 12). Notably, this increase was observed consistently over time. Furthermore, progressors showed higher expansion of LAG3 + helper T cells at 15 months compared to durable responders (*P* = 0.013 estimated by Wilcoxon test; Fig. 5C; Supplemental Table 12). In addition, progressors had a higher proportion of Gr-MDSC at 15 months (*P* = 0.0025 estimated by Wilcoxon test; Fig. 5B; Supplemental Table 12). Conversely, the durable responders showed enrichment of helper T cells (*P* = 0.046 at 6 months) and Vγ9 Vδ2 T cells at different time points (i.e., *P* = 0.048 at 3 months and *P* = 0.008 at 12 months estimated by Wilcoxon test; Fig. 5B; Supplemental Table 12). These findings indicate that progression after Dara-Rd in patients with RRMM is associated with distinct changes over time in the immune compositions with immunosuppressed and immune-exhausted patterns associated with progression.

### Interplay between intrinsic-tumor and extrinsic-immune features

**Fig. 6:**
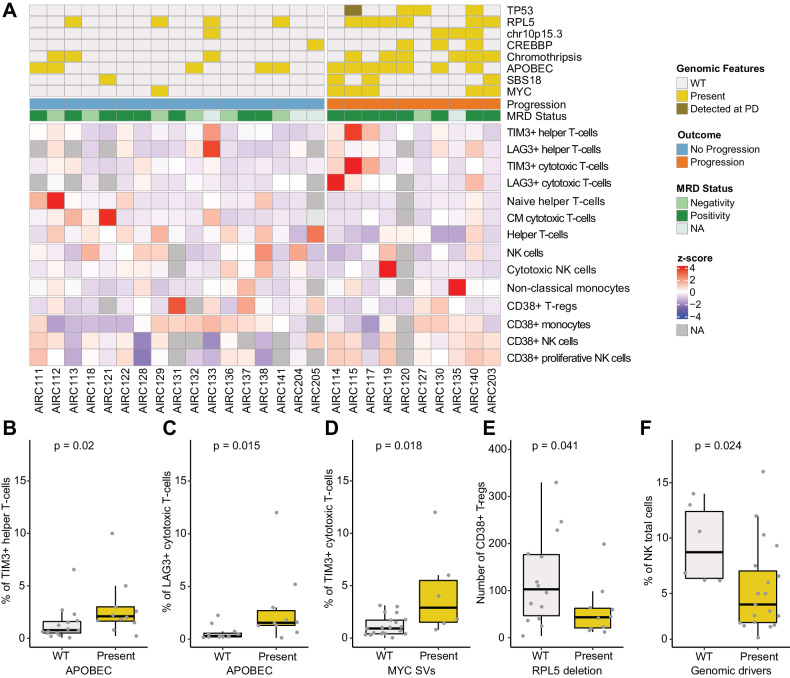
Association between intrinsic-tumor drivers and microenvironment cell populations. **A** Heatmap showing the BM immune cell composition at baseline that were significantly associated with genomic drivers and outcomes. One patient (AIRC123) was excluded because without baseline BM sample. **B**–**F** Boxplots showing the distribution of the immune cells according to the presence of absence of distinct genomic drivers linked to shorter event-free survival (EFS). The color legend is the same of (**A**). All *P* values were computed using Wilcoxon test two-sided.

Because our findings showed that both genomics drivers and immune patterns can influence clinical outcomes in RRMM treated with Dara-Rd, we integrated WGS and flow cytometry data to define possible links between the two compartments. Integrating significant genomic features and distinct immune cell population in 28 patients at baseline, significant associations were observed (Fig. 6A and Supplemental Table 13). Specifically, patients with high APOBEC mutational activity were characterized by an enrichment of exhausted TIM3 + helper T cells (*P* = 0.02; Fig. 6B), and exhausted cytotoxic T-cell expressing *LAG3* and *TIM3* transmembrane protein (*P* = 0.015 and *P* = 0.048 estimated by Wilcoxon test, respectively; Fig. 6C and Supplemental Fig. 7E). Patients without reactive oxygen species damage mutational activity (SBS18) were enriched in cytotoxic NK cells (*P* = 0.044 estimated by Wilcoxon test; Supplemental Fig. 7F). and CD38 pos monocytes (*P* = 0.036 estimated by Wilcoxon test; Supplemental Fig. 7G). SVs involving *MYC* were associated with exhausted TIM3 + cytotoxic T cells (*P* = 0.018 estimated by Wilcoxon test; Fig. 6D) and have less naïve (CD62L + CD45RA + ) helper T cells (*P* = 0.017 estimated by Wilcoxon test; Supplemental Fig. 7H). Patients with 10p15.3 deletions showed high LAG3+ helper T cells (*P* = 0.024 estimated by Wilcoxon test; Supplemental Fig. 7I) and central memory (CD62L + CD45RA-) cytotoxic T cells (*P* = 0.048 estimated by Wilcoxon test; Supplemental Fig. 7J). In addition, deletions involving 1p22.1 (*RPL5)* and the presence of chromothripsis were associated with low CD38 pos T-reg cell levels (*P* = 0.041 estimated by Wilcoxon test; Fig. 6E) and an expansion of nonclassical monocytes in progressed patients with RRMM (*P* = 0.015 estimated by Wilcoxon test; Supplemental Fig. 7K). Overall, reduced baseline number of NK cells was observed in all genomic features associated with progression (*P* = 0.024 estimated by Wilcoxon test; Fig. 6F). Overall, this integrated analysis revealed an association between distinct genomic and distinct immune patterns, which contributed to promote resistance to Dara-Rd. On the other hand, non-progressed patients show a less impaired genome and a less exhausted and immunosuppressed immune composition.

## Discussion

The advent of immune active therapies has significantly improved clinical outcomes in patients with RRMM [3–9]. Despite these improvements, it is unknown why a large fraction of patients fail these new therapeutic approaches. To comprehensively characterize the intrinsic and extrinsic mechanisms of resistance to the daratumumab-based regimen, we integrated WGS data from the tumor cells and flow cytometry of the BM and PB immune composition in 32 patients with RRMM. Thanks to an extensive longitudinal collection of 202 PB samples, we were able to track over time how Dara-Rd affect immune composition and how this was associated with early progression. Despite the relatively limited sample size, our data revealed that RRMM patient progression after Dara-Rd is driven by an intricate interplay between tumor intrinsic genomic features and changes in the extrinsic-immune TME. In line with prior evidence in NDMM patients treated with and without Daratumumab, high APOBEC mutational activity, chromothripsis events, *TP53* deletions and 1p22.1 deletions (*RPL5*), were associated with worse outcomes [14, 18, 26, 33, 36, 40]. *RPL5* loss, recently associated with adverse outcomes after Dara-KRd [33] and VRd [18], has been linked to resistance to proteasome inhibitors due to its biological role in protein transport and regulation [36]. Interestingly, in this study, *RPL5* was associated with a shorter EFS in patients treated without PIs, suggesting a broader role in promoting resistance to various anti-MM therapies. Interestingly, we found SVs involving *MYC* locus were correlated with early progression. Likewise, *MYC* was recently associated with early progression after Dara-KRd treatment in NDMM patients [33]. Moreover, we identified two novel deletions linked to resistance, involving 16p13.3, *CREBBP* tumor suppressor genes, and 10p15.3, both involved in the dysregulation of critical specific pathways, such as *E2F*, *MYC* targets and *G2M* checkpoint pathway that have reported to be enriched in MM that fails daratumumab-based regimens [13, 33]. Finally, these genomic features, particularly APOBEC, have been linked to distinct microenvironment exhaustion, genomic instability, immune escape and inflammatory profile. In summary, our data demonstrate the influence of genomic complexity on the response to modern immunotherapies, highlighting how WGS can comprehensively characterize this complexity and guide the development of personalized treatment strategies.

Through the application of a longitudinal design, we confirmed and expanded the notion that immune patterns at baseline and their changes over time are strongly associated with response and clinical outcomes in MM receiving daratumumab-based regimens [15, 33, 39]. Overall, we found three key immune patterns. The first involves NK cells and the ADCC daratumumab mechanisms of action. Specifically, patients who progressed showed a higher level of CD38 pos NK cells at baseline, which was rapidly depleted as soon as they received the anti-CD38 MoAb treatment. Moreover, in all patients cytotoxic NK cells (CD56 + CD16 + ) develops a drastic reduction after daratumumab treatment, while the proliferative NK cells (CD56 + CD16-) expanded after Dara-Rd treatment in the entire cohort [41]. The increase was primarily driven by CD38 neg proliferative NK cells and was notably higher among durable responders. Moreover, in durable responders, cytotoxic NK cells expanded more at 6 months compared to progressors. This scenario reveals how a less activated immune environment retains a higher pool of NK cells, non-depleted by daratumumab exposure. These NK cells subsequently expand, significantly contributing to the anti-tumor immune activity of daratumumab. Conversely, a more activated immune environment dominated by CD38 pos NK cells, experiences major NK cells depletion after daratumumab, limiting subsequent anti-tumor activity. The second pattern involves FOXP3 + T-reg cells that expand over time, in particular among progressors. The persistence and expansion of these cells can promote an immunosuppressed environment, affecting daratumumab-mediated immune activity against MM. The third pattern revealed how the presence and persistence of exhausted T cell at baseline associated with worse clinical outcomes, in line with what observed with other immunotherapy agents in RRMM [42, 43]. However, a potential limitation of our study is the insufficient number of events for a thorough analysis of rare immune populations.

Overall, the study presented here provides novel insights that contribute to our understanding of resistance to emerging anti-CD38 monoclonal antibodies in MM. Furthermore, it underscores the critical need for integrated multi-omics approaches to thoroughly analyze the tumor cell and immune heterogeneity in MM. This understanding will be instrumental in redefining the concept of high-risk MM and enhancing patient management through the application of personalized medicine.

### Supplementary information

Supplemntary Figures

Supplemntary Methods

Supplemental Tables

## Data Availability

These data have been submitted to EGA under accession numbers EGAD00001010161 (whole-genome sequencing). The Kydar scRNA-seq data are publicly available at the National Center for Biotechnology Information’s Gene Expression Omnibus (accession no. GSE161195). The CoMMpass data was downloaded from the MMRF researcher gateway portal (https://research.themmrf.org). CoMMpass raw data are accessible on dbgap: phs000748.v1.p1.
