# Supplementary material for: Genomic and immune determinants of resistance to daratumumab-based therapy in relapsed refractory multiple myeloma
Source: Blood Cancer J. 2024 Jul 19;14(1):117. doi: 10.1038/s41408-024-01096-6 (PMC11271515; doi:10.1038/s41408-024-01096-6)
Supplement: Supplementary file 1 — Supplemntary Figures [file 41408_2024_1096_MOESM1_ESM.docx]

**Genomic and immune determinants of resistance to anti-CD38 monoclonal antibody-based therapy in relapsed refractory multiple myeloma.**

# **SUPPLEMENTAL FIGURES**

**Supplemental Figure 1. Genomic landscape of 28 patients treated with Dara-Rd. A)** Heatmap showing the 31 MM driver mutated genes among 28 Dara-Rd. Two additional patients treated with other Dara-based regimens were included as well. **B)** The cumulative copy-number plot (top) and cumulative structural variant plot (bottom) across the entire series.


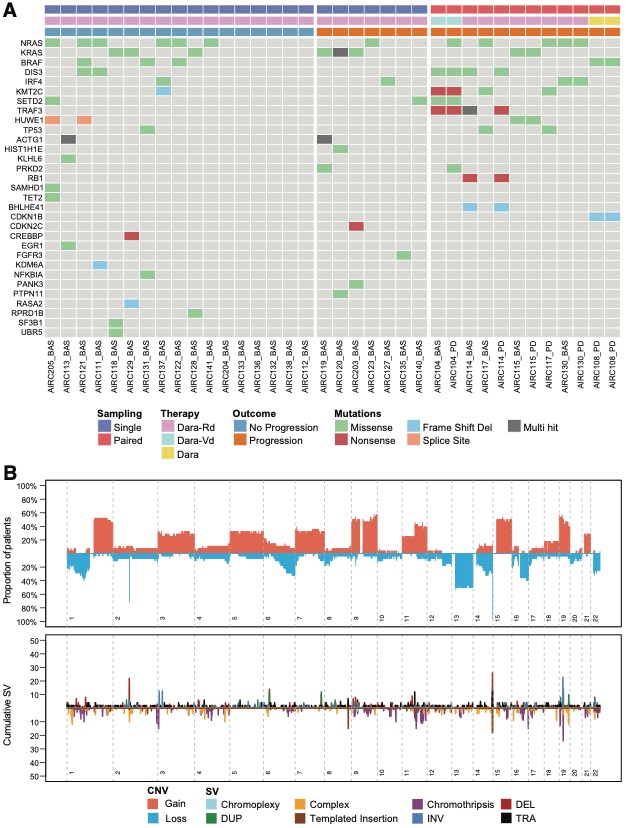


**Supplemental Figure 2. Gene Set Enrichment Analysis (GSEA) for two aneuploidies associated with outcome, using the CoMMpass public dataset.** Heatmap showing the 52-hallmark gene-sets tested in this analysis. The X and * indicate FDR<0.05 and FDR<0.1 respectively, while the bold pathways are significant in both aneuploidies and in the Kydar single cell RNA data set. NES: normalized enrichment score.

**Supplemental Figure 3. Correlation analysis between BM and PB immune composition at baseline. A)** Heatmap showing the Spearman coefficient per each contrast. The color scale is between 0.5 and 1 (red) and -0.5 and -1 (blue). **B)** Scatterplot with the linear regression line (red) between all the number of cells for each immune population in BM and PB at baseline. **C)** Scatterplot using relative values.

**Supplemental Figure 4. Immune composition of RRMM at baseline. A)** Barplot showing the percentage of CD38 pos for each immune cell population. **B)** Barplots showing the percentage of CD38 pos Gr-MDSC and Mo-MDSC cells in BM and PB samples. **C)** The percentage of Gr-MDSC and Mo-MDSC cells in BM and PB samples at baseline. **D)** Barplots presenting the percentages of CD38 pos monocyte subgroups. **E)** The percentage of Monocyte cells at baseline in both BM and PB samples. **F)** Barplot showcasing the percentage of CD38 pos NK cell at baseline in both BM and PB samples. **G)** The percentage of different NK subgroup cells at baseline in both BM and PB samples. The p-values were estimated using Wilcoxon test two sided; *≤0.05, **≤0.01, ***≤0.001, ****≤0.0001. NonCl: non classical; Int: Intermediate.


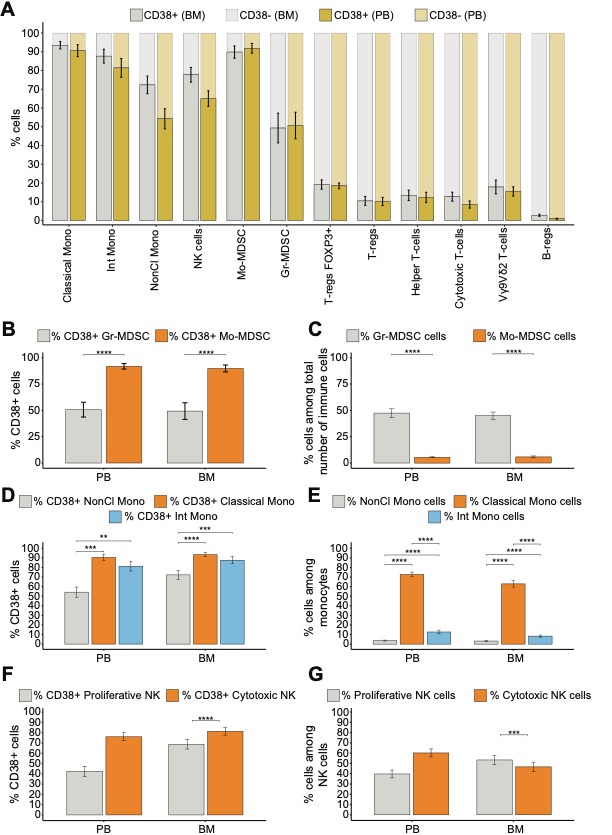


**Supplemental Figure 5. Association between immune composition at baseline with outcome. A-B)** The percentage of CD38 pos immune cell in durable responders and progressors in BM (**A**) and in PB (**B**) samples. **C)** The MFI of CD38 pos among NK cells at baseline in BM. **D-K)** Boxplots showing the associations between immune cells at baseline in BM and PB samples and outcome. All p-values were estimated using Wilcoxon test two sided.

**Supplemental Figure 6. CD38 pos cells and CD38 expression/protein pre and post treatment. A)** Bubble chart about the percentage of CD38 pos immune cell in BM samples before and after Dara-Rd in patients who achieved a durable response. **B)** Boxplots showing the absolute number of Natural Killer cells pre (T1; pink) and post (T2; yellow) Dara-KRd treatment. Data were generated using CITEseq. **C-D)** Boxplots showing the CD38 mRNA expression (**C**) and protein (**D**) (in log scale, post scaling and normalization) in Natural Killer cells pre (T1; pink) and post (T2; yellow) Dara-KRd therapy (Maura et al. Nature Cancer 2023). All p-values were estimated using Wilcoxon test two sided.

#
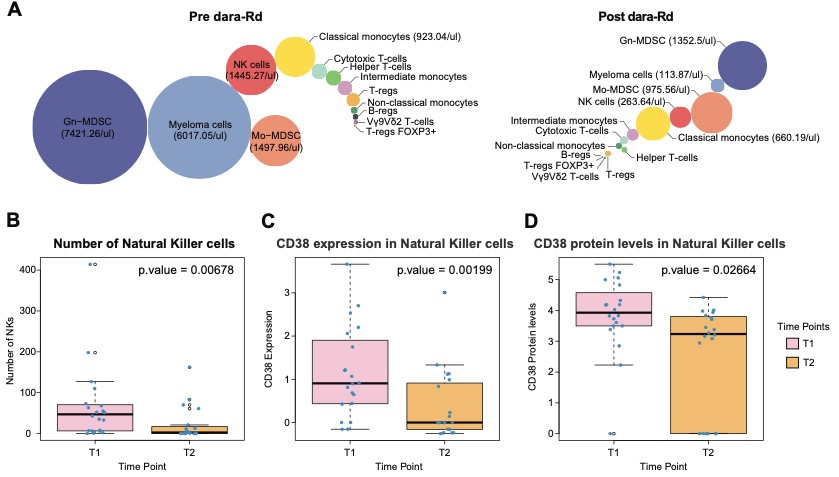


**Supplemental Figure 7. Immunomodulation overtime. A)** Boxplots showing the percentage of CD38 pos intermediate monocyte overtime. **B-C)** Boxplots showing the absolute number of intermediate and non-classical monocytes overtime and based on response to Dara-Rd treatment (durable responders are in blue and progressors in orange). **D)** Boxplots showing the number of CD38 pos CD4+ CD25 CD127low T-reg cells overtime. **E-K)** Boxplots showing the immune cells associated with the significant genomic drivers. All p-values were estimated using Wilcoxon test two sided. The entire list of p-values is available in the **Supplemental Tables 11-12**.

#
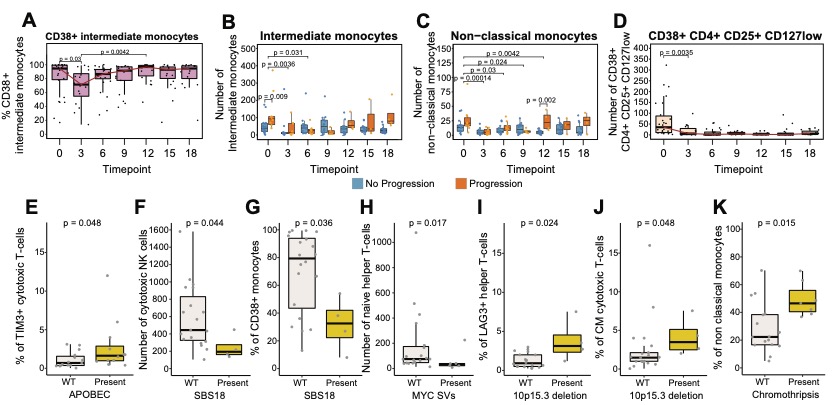


**Supplemental Figure 8. Gating strategy. A)** Gating strategy for CD38 pos NK cells. **B)** Strategy for TIM3+ helper T cells and LAG3+ cytotoxic T cells. **C)** Other phenotypes.
