# Supplementary material for: Genomic and immune determinants of resistance to daratumumab-based therapy in relapsed refractory multiple myeloma
Source: Blood Cancer J. 2024 Jul 19;14(1):117. doi: 10.1038/s41408-024-01096-6 (PMC11271515; doi:10.1038/s41408-024-01096-6)
Supplement: Supplementary file 2 — Supplemntary Methods [file 41408_2024_1096_MOESM2_ESM.docx]

**Genomic and immune determinants of resistance to anti-CD38 monoclonal antibody-based therapy in relapsed refractory multiple myeloma.**

# **SUPPLEMENTAL METHODS**

***Patients***

Thirty-two relapsed/refractory MM patients were enrolled after informed consent and treated with Dara-Rd therapy (NCT03848676). Summary of key clinical and treatment data are available in **Table 1** and **Supplemental Table 1**. The study was conducted in accordance with the Declaration of Helsinki.

***Sample processing***

CD138-positive and germline cells were collected from bone marrow (BM) and (PB) diagnostic samples, respectively. Briefly, Bone marrow plasma cells were enriched using anti-CD138-coated magnetic microbeads and AutoMACS Pro Separator (Miltenyi Biotec GmbH, Bergisch Gladbach, Germany) as previously described (Oliva S Ann Hematol 2021). Purity was assessed by multiparameter flow cytometry (plasma cell purity always exceeded 90%). Differently, germline cells were separated using Ficoll density stratification followed by red cells lysis procedures. Finally, both samples were stored at −80 °C until genomic DNA extraction.

***DNA extraction***

CD138-positive and germline DNA were extracted using commercially available kit Maxwell RSC Blood Kit (Promega, Madison, USA) following manufacturer’s instructions. DNA quantification was performed employing both photometric (NanoDrop/Thermofischer) and fluorescent systems (Quantus; Promega, Madison, USA). 260/280 rand 260/230 ratios were employed to estimate DNA quality.

## ***Whole genome sequencing***

Thirty-two matched tumor-normal samples underwent whole genome sequencing (WGS) at the San Raffaele Institute. In short, libraries were prepared using the Illumina Nextera DNA Flex protocol. Samples were run on NovaSeq 6000 in a 2x150 bp paired end (Illumina).

## ***Whole genome sequencing analysis***

The pre-processing steps and the variant file generation have been performed at San Raffaele Institute. The nf-core/sarek pipeline (<https://github.com/nf-core/sarek>) was used to align tumor and normal paired samples against the GRCh38 reference genome using the Burrows–Wheeler Aligner. All samples were uniformly analyzed by the following bioinformatic tools: somatic mutations were identified by integrating MuTect2 and Strelka2, selecting the variants called by both callers; copy number analysis and tumor purity (i.e., cancer cell fraction) were evaluated using ASCAT; structural variants were defined by Manta algorithm. Complex events (i.e., templated insertions, chromothripsis, and chromoplexy) were defined after manual inspection, as described previously (1). To determine the tumor clonal architecture, we used PyClone algorithm (v0.13.1; <https://github.com/Roth-Lab/pyclone>).

## ***Mutational signatures***

Mutational signatures were analyzed across all whole genomes. To estimate the activity of mutational signatures, we first used a three-step approach of de novo extraction, assignment, and fitting (2, 3). For the first step, we ran SigProfiler for SBS signatures. All extracted signatures were then compared with the latest Catalogue of Somatic Mutations in Cancer (<https://cancer.sanger.ac.uk/cosmic/signatures/SBS>; COSMIC) to identify the known mutational processes active in the cohort. Finally, we applied mmsig (<https://github.com/UM-Myeloma-Genomics/mmsig>) (3), a fitting algorithm, to confirm the presence and estimate the contribution of each mutational signature in each sample guided by the catalog of signatures extracted for each individual sample adding the chemotherapy SBS-MM1 (SBS99) signature for patients who received melphalan (4).

## ***RNAseq analysis and Gene Set Enrichment analysis (GSEA)***

The raw count data was filtered to remove genes with less than 10 reads in greater than 95% of samples. The trimmed mean of M-values (TMM) normalization was applied (5).This method estimates a scale factor used to reduce technical bias between samples with different library sizes. The voom transformation was performed to convert row counts in log2-counts per million (log2-CPM) and calculated the respective observation-level weights to be used in differential expression analysis (6). P values were corrected for multiple testing using Benjamini-Hochberg false discovery rate method. The Gen Set Enrichment Analysis (GSEA) was performed using the *fgsea* R package (7). The H Hallmark gene sets collection, retrieved from MSigDb database v7.4 (8), was enriched with two INF signatures, ISG.RS and IFNG.GS, previously described to be associated with response to immunotherapy (9). Genes were ranked using the statistic derived from differential expression analysis with voom/limma pipeline (6).

***Analysis of malignant plasma scRNA in the Kydar study***

scRNAseq analysis was performed on CD138^+^/CD38^+^ FACS-sorted plasma cells from a previously published Kydar study of 41 patients with MM who failed to respond to a bortezomib-containing induction regimen and received Dara-KRd (10). Gene expression matrices were normalized, scaled, and integrated using Seurat v4 and cells with less than < 500 unique molecular identifiers, < 250 genes, or > 20% mitochondrial genes were excluded. Automated cell classification was performed using *SingleR* with the Human Primary Cell Atlas Dataset serving as reference (11). To better define the malignant plasma cell population, we used *inferCNV* to call copy number variants (CNV) in B cells using non-B cells as the germline reference (https://github.com/broadinstitute/inferCNV). The average expression per sample was computed using the *AverageExpression* function in Seurat. We conducted a differential expression analysis comparing responders and non-responders. Subsequently, we applied Gene Set Enrichment Analysis (GSEA) using the *fgsea* R package, utilizing the same gene sets as previously described. Genes were ranked based on the statistics obtained from the differential expression analysis with the Wilcoxon test, and we applied p-value corrections for multiple testing using the Benjamini-Hochberg false discovery rate method.

***Analysis of NK cell scRNAseq and cyTOF data***

Single-cell data processing followed the outlined guidelines documented in a previous publication (12), ensuring methodological consistency and reliability. To assess the CD38 expression and protein values for each patient, we computed the mean CD38 expression across all NK cells within each patient's sample. Subsequently, we employed a paired Wilcoxon test to analyze potential differences between baseline and post-treatment conditions.

## ***Flow cytometry***

PB and BM samples were collected in heparinized tubes at baseline and at specified time points during treatment [baseline, at follow up every 3 months during therapy (PB only), response to therapy and progression disease (PD)]. White Blood Cells (WBC) obtained after red blood cell lysis were evaluated using real-time flow cytometry or frozen in a solution of FBS containing 10% DMSO.

The majority of samples were analyzed fresh within 24 hours of collection. Only 16% of samples were frozen in liquid nitrogen and processed later. However, thawed samples were incubated for 1h at 37°C to restore the expression of surface molecules before the staining in order not to affect flow cytometric analysis. The comparison between fresh and frozen samples at baseline and 3-month follow up (i.e. timepoint with the highest number of frozen samples n= 10) did not show any significant pattern associated to thawed samples (**Methods Figure 1**).

Immune phenotyping and quantification of T-cells, B-cells, monocytes, Vγ9Vδ2 T-cells, NK cells, MDSC, T-regs, and B-regs were performed serially on BM and PB samples. Cells were washed once with a solution containing PBS and 1% FBS before staining. For membrane immunofluorescence, the cells were incubated with their respective anti-human monoclonal antibodies (mAbs) at 4 °C in the dark for 30 minutes. Afterward, the cells were washed twice and fixed with para-formaldehyde-PBS 1% before the cytofluorimetric acquisition. Cell surface and intracellular proteins were targeted with fluorescinated isocyanine (FITC), r-phycoerythrin (PE), chlorophyll protein peridine (Per-CP) or allophycocyanin (APC), conjugated with mAb, according to the list in **Supplemental Table 14**. For FOXP-3 intracellular staining, cells were fixed and permeabilized using the FoxP3 Staining Buffer Set according manufacturer instructions (Miltenyi #130-093-142). Data were acquired with FACS Calibur cytometers and analyzed using FlowJo software as previously reported. The use of the 4-color FACS Calibur cytometer has significantly limited the number of markers that can be analyzed in a single tube. Therefore, we designed a panel consisting of a large number of tubes (12) in which there are some backbone marker overlaps to identify distinct cell populations (for example T cell markers for tubes 1-4) and differences in the markers for characterization of CD38 expression levels, activation/exhaustion state etc. Thus, we provided a detailed characterization of the several immune populations of interest in the immunomodulatory activity of Daratumumab.

Minimal residual disease was measured by Next generation flow cytometry (NGF), according to Euroflow guidelines (13). The gating strategies for CD38 pos NK cells and TIM3+ helper T-cells and LAG-3+ cytotoxic T cells are shown in **Supplemental Figure 8A-C**. Considering that in-depth analysis of rare populations needs a large number of acquired events, based on *Cossarizza et al*. (14), sufficient event number can be estimated by using Poisson statistics and by keeping CV below 5%. Considering small populations such as TIM3+ T cells and LAG3+ T cells that account for an average of 3- 0.1% of total events, the average number of WBC events acquired in tubes 1-5 (**Supplemental Table 15**) allows to keep CV below 5%.

## ***Statistical analysis***

The comparison tests of the immune-cell types in different groups at different time points was performed with Wilcoxon test two sided. All p-values are two sided if not specified otherwise. To assess the concordance between BM and PB immune cell composition, we performed a correlation analysis (Spearman coefficient) between any BM immune feature and its PB counterpart. Association of categorical variables with EFS were performed in a univariable fashion, using Kaplan-Meier curves and a log-rank test. We conducted a multivariate analysis using the Cox proportional hazards model to assess the independence of each genomic driver found to be significant in univariate analysis from both High-Risk Cytogenetic Abnormalities (HRCA) and International Staging System (ISS) classification. Our approach involved building a base model incorporating HRCA and ISS, and subsequently integrating each significant driver individually, resulting in a total of seven distinct models. The driver DelTP53 was excluded from the analysis due to its inclusion within the HRCA variable. To accommodate the limited number of patients, the ISS classification was re-formatted to distinguish ISS3 group from ISS1 and ISS2 group. EFS was measured from the date of start of treatment to the date of progression. Deaths from causes other than progression were censored.

**REFERENCES**

1. Rustad EH, Yellapantula VD, Glodzik D, Maclachlan KH, Diamond B, Boyle EM, et al. Revealing the impact of structural variants in multiple myeloma. Blood Cancer Discov. 2020;1(3):258-73.

2. Maura F, Degasperi A, Nadeu F, Leongamornlert D, Davies H, Moore L, et al. A practical guide for mutational signature analysis in hematological malignancies. Nat Commun. 2019;10(1):2969.

3. Rustad EH, Nadeu F, Angelopoulos N, Ziccheddu B, Bolli N, Puente XS, et al. mmsig: a fitting approach to accurately identify somatic mutational signatures in hematological malignancies. Commun Biol. 2021;4(1):424.

4. Maura F, Weinhold N, Diamond B, Kazandjian D, Rasche L, Morgan G, Landgren O. The mutagenic impact of melphalan in multiple myeloma. Leukemia. 2021:1-6.

5. Robinson MD, Oshlack A. A scaling normalization method for differential expression analysis of RNA-seq data. Genome Biol. 2010;11(3):R25.

6. Law CW, Chen Y, Shi W, Smyth GK. voom: Precision weights unlock linear model analysis tools for RNA-seq read counts. Genome Biol. 2014;15(2):R29.

7. Subramanian A, Tamayo P, Mootha VK, Mukherjee S, Ebert BL, Gillette MA, et al. Gene set enrichment analysis: a knowledge-based approach for interpreting genome-wide expression profiles. Proc Natl Acad Sci U S A. 2005;102(43):15545-50.

8. Liberzon A, Subramanian A, Pinchback R, Thorvaldsdottir H, Tamayo P, Mesirov JP. Molecular signatures database (MSigDB) 3.0. Bioinformatics. 2011;27(12):1739-40.

9. Jain MD, Zhao H, Wang X, Atkins R, Menges M, Reid K, et al. Tumor interferon signaling and suppressive myeloid cells are associated with CAR T-cell failure in large B-cell lymphoma. Blood. 2021;137(19):2621-33.

10. Cohen YC, Zada M, Wang SY, Bornstein C, David E, Moshe A, et al. Identification of resistance pathways and therapeutic targets in relapsed multiple myeloma patients through single-cell sequencing. Nat Med. 2021;27(3):491-503.

11. Aran D, Looney AP, Liu L, Wu E, Fong V, Hsu A, et al. Reference-based analysis of lung single-cell sequencing reveals a transitional profibrotic macrophage. Nat Immunol. 2019;20(2):163-72.

12. Maura F, Boyle EM, Coffey D, Maclachlan K, Gagler D, Diamond B, et al. Genomic and immune signatures predict clinical outcome in newly diagnosed multiple myeloma treated with immunotherapy regimens. Nat Cancer. 2023.

13. Flores-Montero J, Sanoja-Flores L, Paiva B, Puig N, Garcia-Sanchez O, Bottcher S, et al. Next Generation Flow for highly sensitive and standardized detection of minimal residual disease in multiple myeloma. Leukemia. 2017;31(10):2094-103.

14 Cossarizza A, Chang H, Radbruch A, Akdis M, Andrae I, Annunziato F, et al. Guidelines for the use of flow cytometry and cell sorting in immunological studies. Eur J Immunol. 2017; 10;47(10):1584-1797.
